# Supplementary material for: Preparation of Highly Porous PAN-LATP Membranes as Separators for Lithium Ion Batteries
Source: Nanomaterials (Basel). 2019 Nov 7;9(11):1581. doi: 10.3390/nano9111581 (PMC6915605; doi:10.3390/nano9111581)
Supplement: Supplementary file 1 [file nanomaterials-09-01581-s001.pdf]

Supplementary Information

Preparation of Highly Porous PAN-LATP Membranes as Separators for Lithium Ion Batteries

Jagdeep Mohanta [1], O Hyeon Kwon [2], Jong Hyeok Choi [1], Yeo-Myeong Yun [3], Jae-Kwang Kim [2,*] and Sang Mun Jeong [1,*]

[1] Department of Chemical Engineering, Chungbuk National University,1 Chungdae-ro, Seowon-gu, Cheongju, Chungbuk 28644, Korea; jagdeepmohanta@gmail.com (J.M.); qweaq1@naver.com (J.H.C.)

[2] Department of Solar & Energy Engineering, Cheongju University, Cheongju, Chungbuk 28503, Korea; ohhyeon2940@naver.com

[3] Department of Environmental Engineering, Chungbuk National University, 1 Chungdae-ro, Seowon-gu, Cheongju, Chungbuk 28644, Korea; ymyun@cbnu.ac.kr

* Correspondence: jaekwang@cju.ac.kr (J.-K.K.); smjeong@chungbuk.ac.kr (S.M.J.)

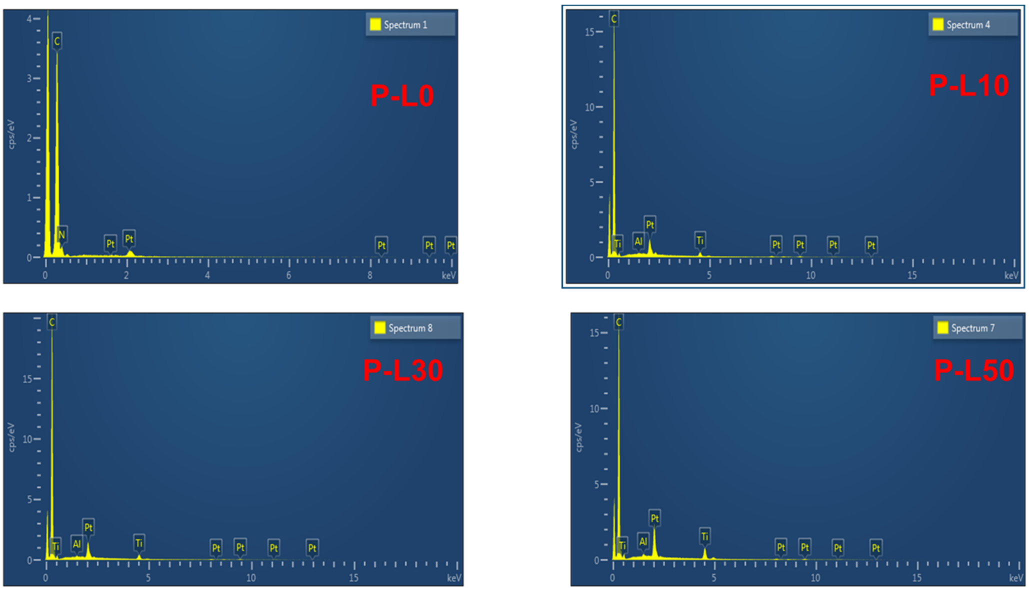

Figure S1. EDS of PL membranes

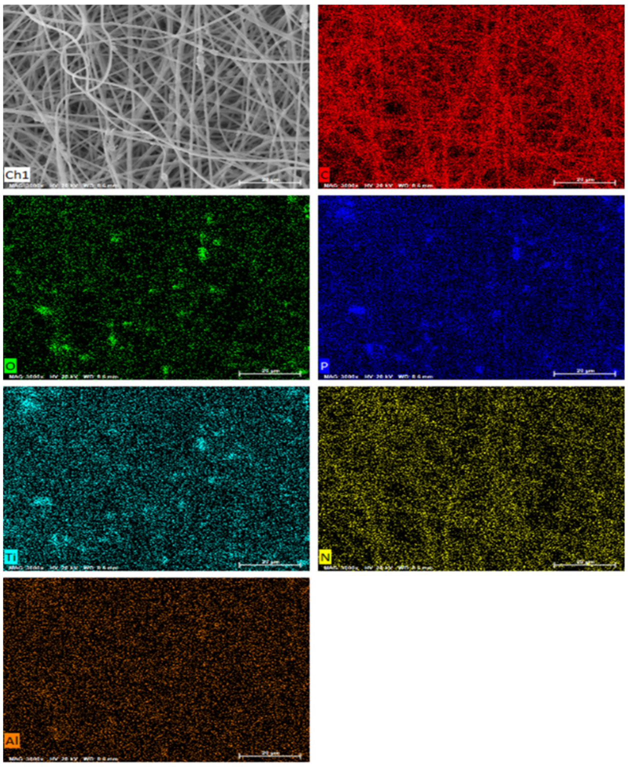

Figure S2. Elemental Mapping of P-L30

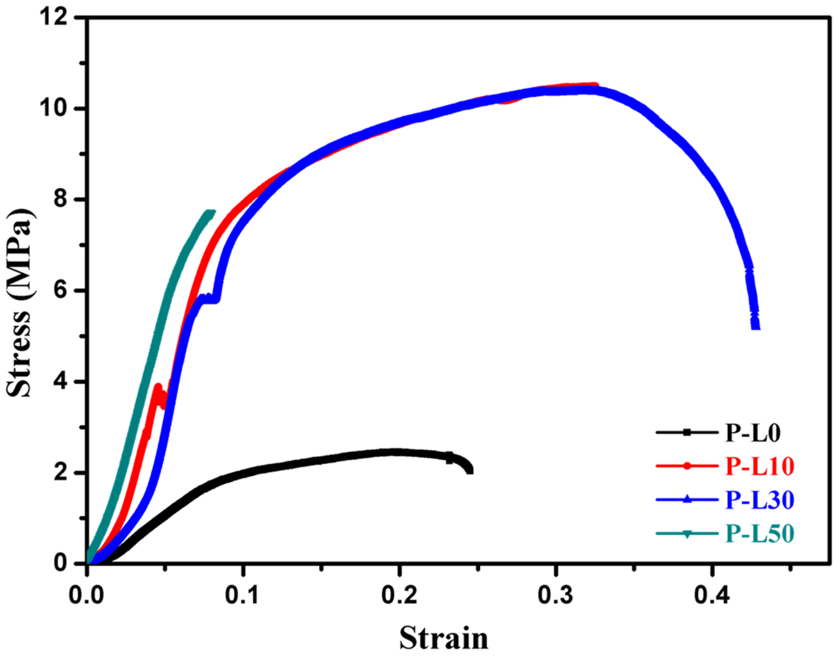

Figure S3. Stress-Strain curves of PL membranes

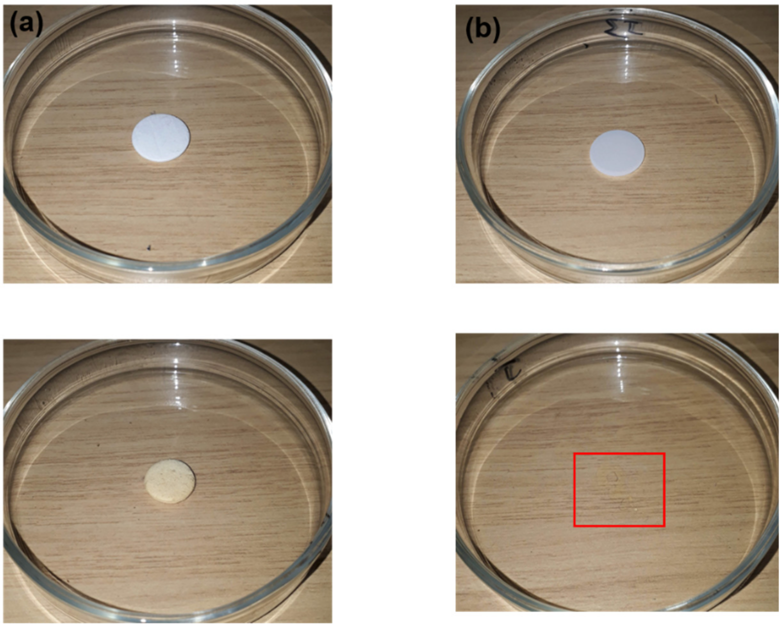

Figure S4. Heat treatment at 170 °C of **(a)** P-L30 **(b)** celgard separator (Above — Before Heat treatment; Below — After Heat treatment)

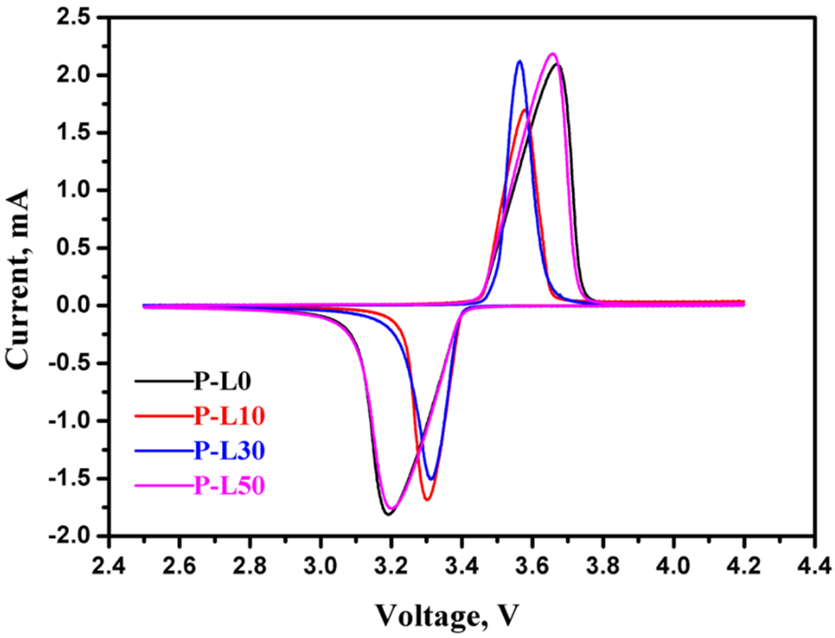

Figure S5. Cyclic Voltammetry of PL membranes

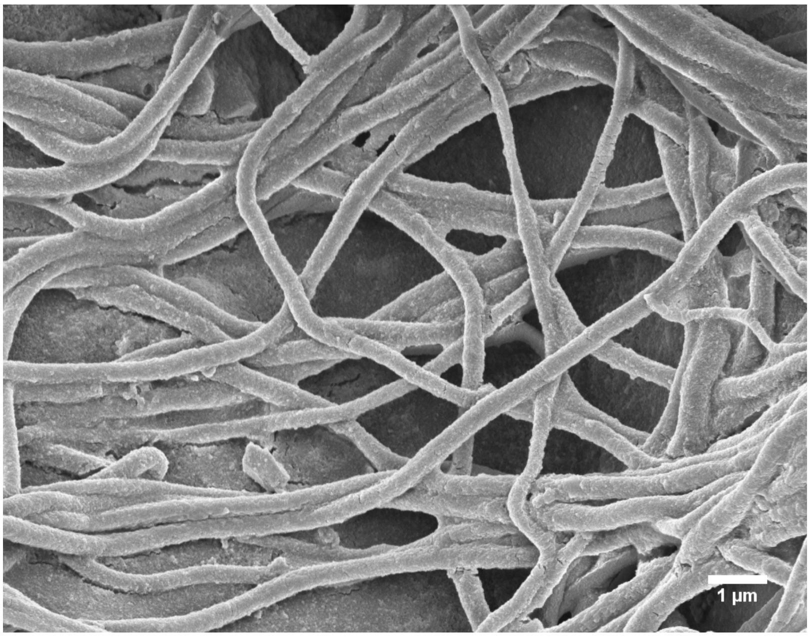

Figure S6. FESEM image of P-L30 membrane after 200 cycles of Charge and discharge
